# Reduced anogenital distance, hematuria and left renal hypoplasia in a patient with 13q33.1–34 deletion: case report and literature review

**DOI:** 10.1186/s12887-020-02205-7

**Published:** 2020-07-02

**Authors:** Xue He, Huijun Shen, Haidong Fu, Chunyue Feng, Zhixia Liu, Yanyan Jin, Jianhua Mao

**Affiliations:** Department of Nephrology, National Clinical Research Center For Child Health, The Children’s Hospital, Zhejiang University School of Medicine, #57 Zhugan Lane, Hangzhou, Zhejiang Province 310003 P.R. China

**Keywords:** 13q deletion syndrome, 13q33–34 deletion, Chromosome 13, Renal hypoplasia, Congenital heart disease

## Abstract

**Background:**

13q33–q34 microdeletions are rare chromosomal aberrations associated with a high risk of developmental disability, facial dysmorphism, cardiac defects and other malformation of organs. It is necessary to collect and report evidence of this rare chromosome mutation to improve the prognosis of this rare disease.

**Case presentation:**

We report a patient harboring an 11.56 Mb microdeletion at 13q33.1–34 region, which contains about 30 OMIM genes. Besides the common clinical manifestations such as facial dysmorphism, developmental delay, intellectual disability, epilepsy, and congenital heart disease, she also suffered from a reduced anogenital distance, hematuria and left renal hypoplasia. Most related cases were characterized by facial deformity and heart defects, but there were few reports on renal malformation, especially regarding renal hypoplasia with hematuria.

**Conclusion:**

We have reported a patient suffering from a reduced anogenital distance, hematuria and left renal hypoplasia. A de novo 11.56 Mb deletion ranging from 13q33.1 to 13q34 (Chr13:103542220–115,106,996) was found by SNP-array analysis. It might be the first time for hematuria and renal hypoplasia to be reported as symptoms of 13q33-q34 deletion syndrome Neurodevelopmental disability, heart defects and urogenital/anorectal anomalies may be resulted from common or overlapping regions of deletion in chromosome bands 13q33.1-q34 and may share a common molecular mechanism.

## Background

13q deletion syndrome is a rare genetic disorder caused by the deletion of the long arm of chromosome 13 [1, 2]. It was first reported in patients with mental and growth retardations in 1963 [3]. Patients with 13q deficiency exhibit a variety of phenotypic characteristics, including intellectual disability, hypotonia, developmental delays, microcephaly, central nervous system abnormalities, microphthalmia, heart defects, urogenital abnormalities, and limb abnormalities [1, 4]. According to the sizes and locations of the deletions, they can be divided into three types: 1) deletions of chromosome regions near the band 13q32; 2) deletions of chromosome band 13q32; and 3) deletions of distal band 13q33–34. 13q deletion syndrome is a rare genetic disorder, especially the third type described above [5]. With the development of chromosomal microarray analysis technology, increasing number of microdeletions are being identified [6]. However, until now, a limited number of cases for 13q33-q34 deletion syndrome were reported in the literature and most related cases were characterized by facial deformity or heart defects [2, 7, 8]. Moreover, there were few reports on renal malformations. Here we described a seven years old girl harboring an 11.56 Mb microdeletion at 13q33–34 region with facial abnormalities, hypotonia, growth delay, psychomotor developmental delay, epilepsy, reduced anogenital distance, hematuria and left renal hypoplasia.

## Case presentation

**Fig. 1:**
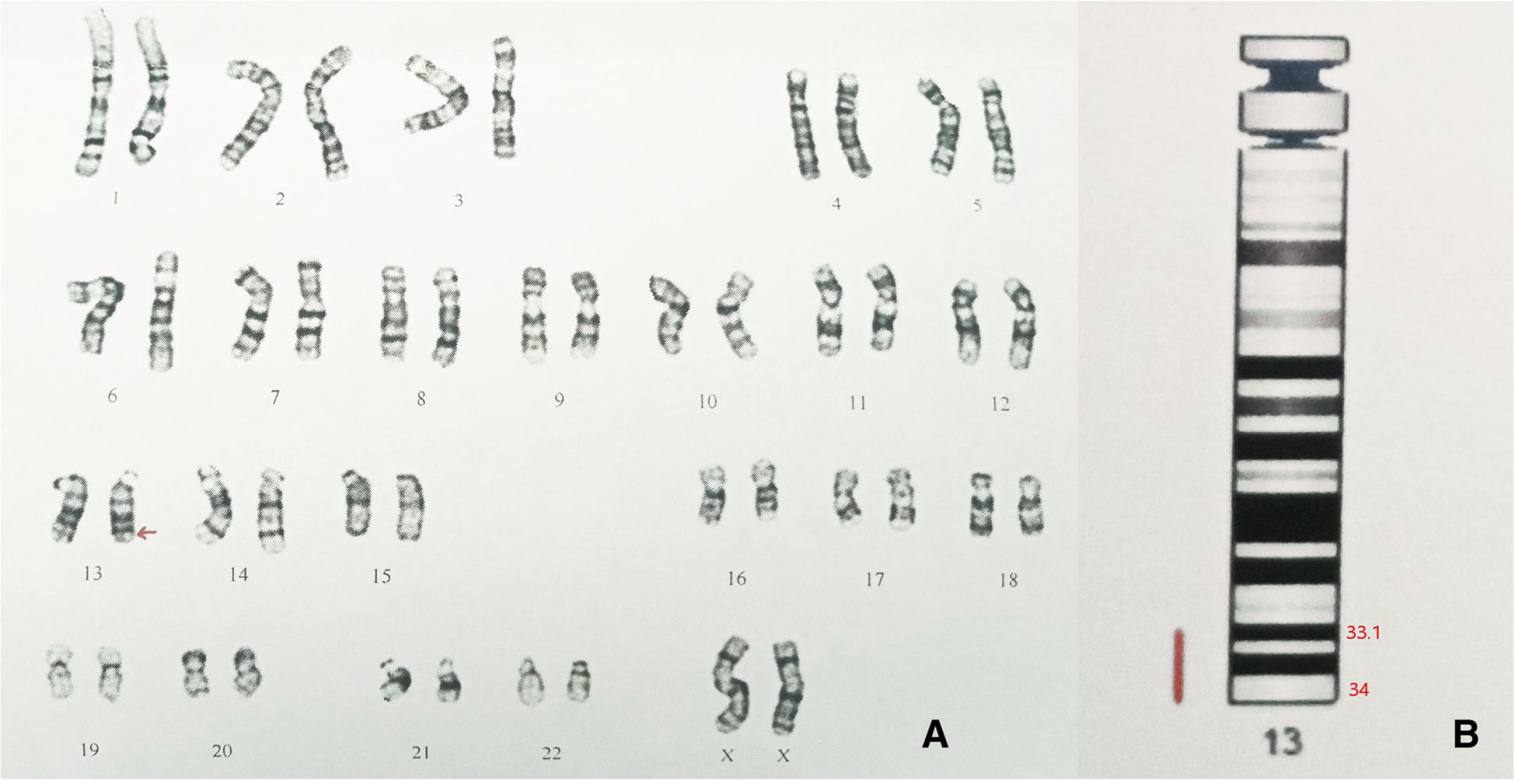
Karyotype chart of the patient. **a** Chromosome analysis by G-banding at 550-band level showed a deletion of the long arm of chromosome 13. **b** SNP-array analysis revealed a de novo 11.56 Mb deletion ranging from 13q33.1 to 13q34

**Fig. 2:**
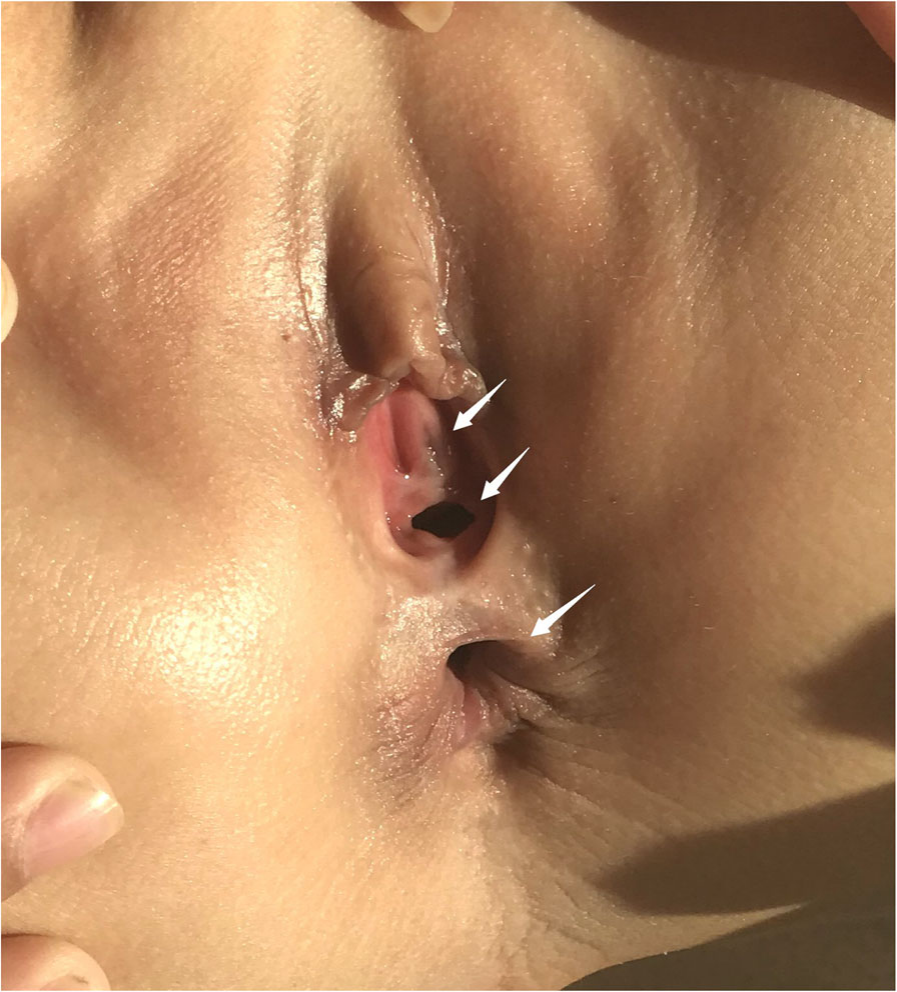
Reduced anogenital distance

**Fig. 3:**
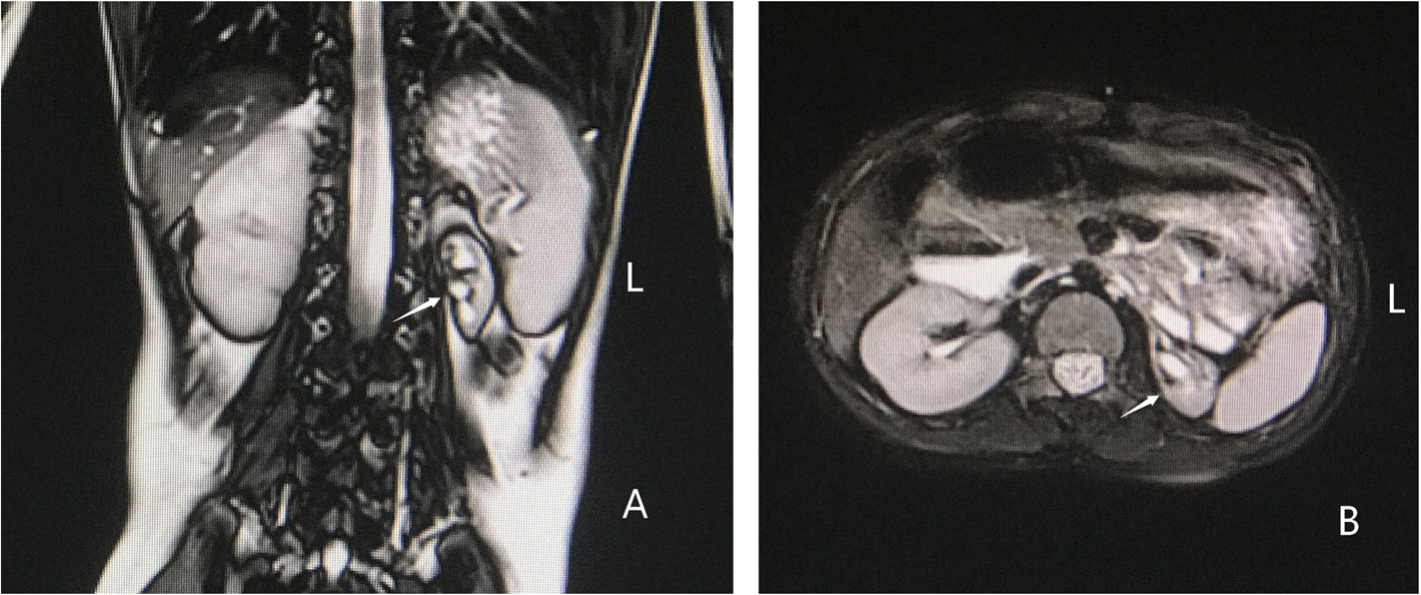
Renal MRI of the patient, viewing the kidney in the coronal plane (**a**) and the transverse plane (**b**). The left kidney was small in size; the boundary between cortex and medulla was not clear; renal pelvis and calyces dilation was detected. The right kidney was normal in shape and size

The patient was a 7-year-old female born at 41 weeks of gestation by normal vaginal delivery. A “Double Bubble” sign was observed by fetal ultrasound at 39 weeks of gestation. Her birth weight was 3050 g. Her parents were healthy and unrelated and her mother denied any history of taking teratogenic drugs, heavy drinking or diabetes during pregnancy. The girl was the first child of the family and had no siblings. She had normal meconium but was found to have hypotonia, weak crying, and facial abnormalities after birth. Muscular ventricular septal defect (2.5 mm) and patent ductus arteriosus (1.4 mm) were detected in the patient by cardiac ultrasound. In addition, the patient failed the hearing screening and the brainstem auditory evoked potential (BAEP) test. Chromosome G-banding analysis was carried out in the neonatal period and showed a normal female karyotype. As she grew up, her motor and mental development significantly lagged behind her peers. Cytogenetic analysis and SNP-array analysis were performed when she was two years old. The chromosome analysis of the girl revealed a deletion of the long arm of chromosome 13 (Fig. 1), described as 46, XX, del (13)(q33.1). Compared with the Database of Genomic Variants (DGV), a de novo 11.56 Mb deletion ranging from 13q33.1 to 13q34 (Chr13:103542220–115,106,996) was found. This chromosome region contains about 30 OMIM genes, including SLC10A2, DAOA, EFNB2, ARGLU1, LIG4, TNFSF13B, IRS2, COL4A1, COL4A2, CARS2, ING1, ARHGEF7, SOX1, ATP11A, MCF2L, F7, F10, PROZ, PCIDI, CUL4A, LAMPA, ADPRHL1, TFDP1, ATP4B, GRK1, GAS6, RASA3, and UPF3A. As she grew up, microscopic hematuria and urinary tract infections were detected by routine urine tests when she suffered from a fever, with her red blood cell count being 20–50/HP. She suffered her first grand mal epileptic seizure when she was 6 years old and was treated with valproate since then. In October 2019, when she was about 7 years old, she was hospitalized in our department and a thorough physical examination was carried out. Her blood pressure was normal, and her height and weight were 120.0 cm (P25) and 20.0 kg (P25) respectively. She had a flat and leaning head, hypertelorism, malformed ears, flat and broad bridge, micrognathia, and transverse palmar crease on both hands. In addition, the distances between her urethra, vagina and anus were shorter than normal (Fig. 2). She had congenital dislocation of the left hip joint and she was incapable of walking independently. Her psychomotor milestones were delayed with mental retardation and she suffered from incontinence. Routine blood, liver function and renal function tests were normal, the tests on complement C3, antinuclear antibody (ANA), antineutrophilic cytoplasmic antibody (ANCA), and HBV test also showed no abnormalities. The test on urinary microprotein was normal with a urine protein/creatinine ratio of 0.16 and a urinary calcium/creatinine ratio of 0.01. During this time, a cardiac ultrasound was performed again but only found mild tricuspid regurgitation with no VSD or PDA. Uterine and ovarian ultrasound scans were normal and renal vein ultrasound showed no symptom of left renal vein entrapment syndrome. Renal ultrasound scans indicated that the left kidney was small with a size of 4.2 cm × 1.9 cm(less than two standard deviations), while the right kidney was 8.2 cm × 4.5 cm in size. The renal MRI showed left renal hypoplasia and hydronephrosis (Fig. 3). Dynamic renal function imaging showed that the glomerular filtration rate (GFR) of the left kidney was 19.34 ml/min and the GFR of the right kidney was 85.81 ml/min. To further investigate the cause of renal miniaturization and hematuria, a renal vascular contrast-enhanced ultrasonography and a contrast-enhanced voiding urosonography (ceVUS) were performed. The results suggested that (1) The left kidney was smaller than normal, and the left renal artery was relatively thin (diameter of the left renal artery was 0.10 cm, while that of the right renal artery was 0.30 cm). The enhancement degree of contrast-enhanced ultrasound of the left kidney was weak compared to the right. (2) The left lower ureter was slightly dilated and a mild vesicoureteral reflux (grade I) of the left side was detected. Nitrofurantoin (1 mg/kg/d was given every night to avoid recurrent urinary tract infection and the patient did not suffer from any urinary tract infection in the past few months. However, microscopic hematuria was detected every time during routine urine tests. Because of the unilateral renal hypoplasia, a kidney biopsy was not performed.

## Discussion and conclusions

Chromosomal microarray analysis (CMA) is a technology comprising array-based comparative genome hybridization and single-nucleotide polymorphism arrays. It is used for the detection of clinically significant microdeletions or duplications with high sensitivity for submicroscopic aberrations [6, 9]. The chromosome analysis of our patient revealed a deletion of the long arm of chromosome 13. The molecular karyotype was described as arr 13q33.1q34(103542220–115,106,996) × 1 by the SNP-array, and the chromosome G-banding analysis showed a normal female karyotype in the neonatal period. Our case once again demonstrated the important role of CMA. We strongly urge patients with facial deformities, developmental delay and multiple congenital anomalies to undergo the CMA test as early as possible, despite getting normal results from the chromosome karyotype tests.

Deletions of chromosome bands 13q33–34 are rare. Majority of patients with such deletions have mental retardation, microcephaly, and distinct facial features [4]. This study reports a patient with a de novo 11.56 Mb deletion ranging from 13q33.1 to 13q34 (Chr13:103542220–115,106,996) which is a chromosome region containing about 30 OMIM genes, including eight OMIM morbid genes: *SLC10A2*, *LIG4*, *COL4A1*, *COL4A2*, *ING1*, *F7*, *F10* and *GRK1*. The patient suffered from a reduced anogenital distance, hematuria and left renal hypoplasia in addition to the common clinical features of mental retardation, facial abnormalities, and congenital heart diseases.

Genitourinary/anorectal anomalies in the 13q deletion syndrome are rare and they vary in severity and manifestation. Anal atresia, hypospadias and perineal fistula were recorded to be observed in male patients [7, 10]. However, only a few cases were identified in females, and they were often misdiagnosed as anal atresia or vaginal fistula [8, 11]. Joanna et al. [8] reported that 13q33–34 might contain a gene for male genital development. The non-morbid OMIM gene ephrin B2 (EFNB2), located in 13q33.3, was recognized recently as a strong candidate gene responsible for hypospadias and anorectal anomalies in 13q deletion syndrome in severl studies [8, 12]. There was no urethrovaginal fistula or any other abnormalities in our patient. The uterine and ovarian ultrasound scans were normal but the patient had a reduced anogenital distance.

There were less than five cases reported with renal malformation [8, 12]. Jonna et al. [8] reported a boy suffering from malformed genitalia (penoscrotal transposition and hypospadias) and his ultrasound examinations showed pelvic displacement of the right kidney. Kuhnle U et al. [12] reported a boy with penoscrotal inversion and hypospadias and his B-mode ultrasound of the urogenital tract revealed the absence of the left kidney. For our patient, urological ultrasound and MRI revealed unilateral renal hypoplasia. Microscopic hematuria was detected by the routine urine tests. The girl did not have any history of acute kidney injury or usage of nephrotoxic drugs during her infancy. Causes of hematuria such as idiopathic hypercalciuria, urolithiasis, left renal vein compression syndrome and urinary tract infections were excluded by detailed clinical examinations. We postulated that the underlying renal abnormality associated with 13q deletion might be the cause of hematuria. We believed that there might be a relation between renal agenesis and 13q33-q34 deletion. This region is likely to contain one or more developmental genes and deletions or haploinsufficiency of these genes can result in genitourinary system malformations. The pathogenesis of renal malformation has not been elucidated but many researchers are paying more attention to gene mutation and copy number variation to be the possible causes In addition to genetic factors, environmental factors during pregnancy can also influence kidney development. These factors involve taking teratogenic drugs during pregnancy, heavy drinking and diabetes [13–15]. Currently, the specific molecular mechanism of renal malformation is still pending further investigation. Our case may be a clue to the mechanism of renal agenesis.

Previously reported cases and studies suggested that 13q33.1–34 deletion was closely associated with congenital heart diseases (CHD) [2, 16, 17] and approximately 50% of the patients had CHD [16]. CHD in 13q deletion syndrome is more complex than in isolated cases and of the complexity comes from the presence of DORV, Tetralogy of Fallot, at least 2 heart anomalies in one patient or rare type complex heart anomalies [2, 7, 16, 17]. The complexity suggests that multiple genes may be involved in its pathogenesis. Huang et al. [2] hypothesized that a 6 Mb region of 13q33.1-q34 may contain a critical region for cardiac development, and some researchers proposed COL4A1 and COL4A2 to be the possible candidate genes. These two OMIM genes may contribute to the development of cardiovascular diseases [2, 7, 18–20]. COL4A1 and COL4A2 are one of three pairs of paralogous genes that constitute type IV collagen. These two genes are ubiquitously expressed in the basement membrane, during early stages of development. Mutations of COL4A1 and COL4A2 are increasingly recognized as causes of multisystem disorders [21]. The spectrum of COL4A1-related disorders includes: 1) brain small vessel disease 1 with or without ocular anomalies, 2) hereditary angiopathy with nephropathy, aneurysms, and muscle cramps, 3) susceptibility to intracerebral hemorrhage, 4) tortuosity of retinal arteries, 5) autosomal dominant pontine microangiopathy and leukoencephalopathy [22]. The spectrum of COL4A2-related disorders includes brain small vessel disease 2 [23, 24] and susceptibility to intracerebral hemorrhage [25]. Environmental factors or other genetic modifications may also influence the phenotypic expression and the severity of the organs’ involvement in the related disease. The studies by Schenke Leyland et al. [26] and Hanson et al. [20] demonstrated that type IV collagen played a vital role in early cardiac development. Nonetheless, there was still no direct evidence that could prove the exact relationship between CHD and these 2 genes. Approximately 50% of patients with deletions of COL4A1 and COL4A2 did not have congenital heart defects but our patient belonged to the other 50% who did suffer from CHD (in her case were VSD and PDA). However, her heart defects healed spontaneously during the process of her development. Hence, we believe that children with chromosome abnormalities can also spontaneously recover from mild congenital heart disease the same way as normal children.

Most children with chromosome disorders also have mental retardation and they usually suffer from micturition incontinence. Therefore one should be aware of the possibility of recurrent urinary tract infections and vesicoureteral reflux. Contrast-enhanced voiding urosonography (ceVUS) is a dynamic imaging technique, which is often indicated to depict vesicoureteral reflux (VUR) [27]. ceVUS has become routine screening for VUR in children in Europe and it was shown to be capable of detecting higher grades of reflux in addition to being more sensitive compared to voiding cystourethrography (VCUG) [27, 28]. However, ceVUS has not been widely used in China. In deference to the wishes of the patient’s parents, we performed this technique and detected slight reflux. In addition, ionizing radiation was avoided.

In conclusion, we reported a patient with a de novo 11.56 Mb microdeletion ranging from 13q33.1 to 13q34 (Chr13:103542220–115,106,996). Besides the common clinical manifestations such as facial dysmorphism, developmental delay, intellectual disability, epilepsy, and congenital heart disease, she also suffered from a reduced anogenital distance, hematuria and left renal hypoplasia. It mighe be the first time for hematuria and renal hypoplasia to be reported as symptoms of 13q33-q34 deletion. Neurodevelopmental disability, heart defects and urogenital/anorectal anomalies may be resulted from common or overlapping regions of deletion in chromosome bands 13q33.1-q34 and may share a common molecular mechanism. However, the molecular mechanism is still not very clear until now. With the development of CMA technology, increasing number of microdeletions are being identified and this will provide a greater understanding of the molecular mechanisms of chromosome 13q deletion syndrome.

## Data Availability

The data of the current study are available from the corresponding author on reasonable request.

## Abbreviations

CMA: Chromosomal microarray analysis
SNP: Single-nucleotide polymorphism
CHD: Congenital heart disease
DORV: Double outlet right ventricle
ceVUS: Contrast-enhanced voiding urosonography
